# Emergency department performances during overcrowding: the experience of the health protection agency of Brianza

**DOI:** 10.3934/publichealth.2018.3.217

**Published:** 2018-06-29

**Authors:** Emanuele Amodio, Luca Cavalieri d'Oro, Elisabetta Chiarazzo, Carlo Picco, Maurizio Migliori, Isabella Trezzi, Silvano Lopez, Oliviero Rinaldi, Massimo Giupponi

**Affiliations:** 1 Health Protection Agency of Brianza (Italy), Viale Elvezia n.2 Monza (MB) 20900; 2 AREU−Urgency Emergency Regional Agency, Lombardy

**Keywords:** emergency department, overcrowding, improvement, mortality

## Abstract

*Background:* Hospital emergency departments (ED) can contribute to improve health outcomes and reduce costs of health care system. This study evaluated ED admissions during a twelve months period, analyzing characteristics of patients who underwent to emergency care in order to understand factors involved in ED overcrowding and promote adequate management. *Methods:* This retrospective study analyzed a twelve months window, with in-depth focus on December/January when almost all EDs reported overcrowding. All ED admissions were recorded in electronic schedules including: demographic characteristics, time/date of the access, incoming triage code, diagnosis, performed procedures, discharge, time/date of discharge. A backward multivariable logistic regression model was used to estimate relationships between investigated variables and ED pattern mortality. *Results:* A total of 416,299 ED admissions were analyzed. During the overcrowded period there was an increase in patients admissions (+32 patients per day, *p* = 0.0079) with a statistically significant rise of critical patients (+1.7% yellow codes and +0.7% red codes, *p* < 0.001) and older subjects (+1.4% patients aged 75 or more years, *p* < 0.001). Moreover, there were statistically significant increases in waiting times and in length of visits, a higher percentage of patients who were hospitalized (13.3% *vs.* 12.2%, *p* < 0.001), left ED (4.46% *vs.* 4.15%, *p* < 0.001) and died (0.27% *vs.* 0.17%, *p* < 0.0001). This latter result maintained a marginal statistical significance (OR = 1.16, 95% CI = 0.98–1.38, *p* = 0.075) after adjustment for confounding. *Conclusion:* Our study highlights that ED crowding can determine measurable worsening in ED services and patient outcomes as mortality, waiting times, lengths of stay, percentage of abandonment without being seen and, probably, costs. Thus, address ED crowding has to be considered an important public health priority requiring policymakers involvement.

## 1. Introduction

Hospital emergency departments (ED) play a key role in the health care system, addressing emergent or urgent health problems, whose treatment often cannot be delayed. Emergency physicians are demanded to provide high quality emergency care to all patients in a safe and timely manner but this goal cannot be reached without a clear and efficient organization of the services.

Moreover, the relevant socio-demographic changes observed in the last decades in developed countries, with an aging population and the spending review, could have significant influences in increasing ED activities.

In particular, there are suggestions that the increment of activity can be in particular in favour of those patients that are described as “frail” and include elders, with several comorbidities and with repeated visits, usually 4 or more per year [1]. Some authors have reported that these challenges have resulted in ED overcrowding, too long waiting times, inefficiencies in providing care, patient discomfort, and longer in-hospital length of stay [2],[3]. Concomitant progressive reduction of inpatient beds has been also reported as contributing factor [4]. Prolonged waiting times and poor health outcomes for patients have been strictly associated with ED crisis. Other important consequences of overcrowding include a general perception of being rushed by emergency physicians and staff and a high frequency of patients leaving the ED with no visit. It should be pointed out that ED utilization could reflect greater health needs of the surrounding community and may provide the only readily available care for individuals who cannot obtain care elsewhere. Thus, many ED admissions could be, at least in part, potentially preventable by offering access to high-quality, community-based health care that could avoid a portion of ED visits.

However, a part of ED admissions are related to patients who self-refer to emergency departments to ask treatment for minor or unexpected illnesses instead of referring to the general practitioner (GP). The percentage of access to ED for non-urgent causes is high worldwide ranging from 24 percent to 40 percent of total accesses [4]. Great efforts have been made in Italy to reduce hospital care costs fulfilling the health needs of the population. There is widespread agreement that structural changes are needed to improve health outcomes for older people and to simultaneously reduce National Health Service costs [5]. Trying to solve ED problems requires the revision of the entire process of ED care: therefore the knowledge in depth of the epidemiologic characteristics of ED admissions should be a priority for public health authorities. Following the previous considerations and in order to answer some unsolved questions, this study evaluates ED admissions during a twelve months period, analyzing characteristics of patients who underwent to emergency care.

## 2. Methods

Using data from the regional health system we carried out a retrospective cohort study on all ED admissions of patients that live in two Italian provinces (Lecco and Monza e Brianza), including 9 Emergency Departments and accounting for a total of 1,205,330 inhabitants (Italian National Institute of Statistics–ISTAT, 2016) [6].

The study included a twelve months window, from 1^st^ September 2016 to 31^th^ August 2017, with in-depth focus on the months of December 2016 and January 2017 (since now defined as “overcrowded period”) when almost all EDs reported overcrowding.

We considered ED crowding according to the American College of Emergency Physicians (ACEP) Crowding Resources Task Force, as a situation in which the defined need for emergency services outstrips available resources in the ED [7].

In Lombardy, each ED admission is recorded and sent to the regional healthcare information system. ED admission information include: demographic characteristics (age, sex, residence), time and date of the access, incoming triage code assigned by nurses and outgoing triage code assigned by physicians (“white”: non-critical patients who should receive primary care; “green”: not life-threatening conditions; “yellow”: critical patients at risk of deteriorating clinical condition; “red”: very critical patient needing immediate treatment), diagnosis (categorized as major diagnostic categories–MDC), performed procedures, type of discharge from ED (classified as patient hospitalized, arrived dead, sent home, sent to another institute, died at the ED, patient refused hospitalization, patient left the ED before being examined or referred for outpatient care), time and date of the discharge. In Italy, access to health care is a tax-financed service, and access to emergency medical service is always free of charge. First aid is completely free of charge for urgent cases while a small amount maybe charged in case of non-urgent visits.

All data were managed and analyzed using the statistical software R system, version 2.15.1 [8]. The significance level was set at *p* < 0.05 (two-tailed). Absolute and relative frequencies were calculated for qualitative variables while quantitative variables were summarized as mean when normally distributed or otherwise as median (interquartile range–IQR). Data normality was verified by the Shapiro–Wilk test for normality. Categorical variables were analysed using chi-squared test (Mantel-Haenszel). Means were compared by using Student t test and medians by the Wilcoxon tests. A backward multivariable logistic regression model was used to estimate relationships between investigated variables (age, sex, triage code and overcrowded period) and ED pattern mortality.

According to Italian law on observational studies that use administrative aggregate data, neither ethics approval nor individual written consent by patients was asked.

## 3. Results

During the twelve months window, a total of 416,299 ED admissions were recorded. Table 1 summarizes characteristics of admissions occurred between December 2016 to January 2017 compared with admissions during other months of the study period.

Overall, during the overcrowded period there was a slight increase in number of patients admissions (+32 patients per day, *p* = 0.0079) with a statistically significant rise of admissions of females (62.7% *vs.* 61.5%, *p* < 0.001), critical/very critical patients (+1.7% yellow codes and +0.7% red codes, *p* < 0.001) and older subjects (+1.4% patients aged 75 or more years, *p* < 0.001).

As reported in Table 2, the overcrowded period was characterized by a statistically significant increase in waiting times (+7 minutes for white codes, +9 minutes for green codes, +3 minutes for yellow codes and +1 minute for red codes) and in length of visits for critical (+13 minutes for yellow codes, *p* < 0.001) and very critical patients (+28 minutes for red codes, *p* < 0.001). Moreover, during the overcrowded period, a higher percentage of patients were hospitalized (13.3% *vs.* 12.2%, *p* < 0.001), left ED before being examined or referred for outpatient care (4.46% vs. 4.15%, *p* < 0.001) and died (0.27% *vs.* 0.17%, *p* < 0.0001). This latter result maintained a marginal statistical significance (OR = 1.16, 95% CI = 0.98–1.38, *p* = 0.075) after adjustment for potential confounding due to sex, age and admission triage codes (Table 3).

**Table 1.** Socio-demographic characteristics of patients admitted at the ED during two different study periods.

	December 2016 to January 2017	Other months of the windows period	*p*-value
Mean daily ED admissions, n (rate×1,000)	1,173 (0.97)	1,141 (0.94)	0.0079 ^a^
Sex, n (% by column)			
*Males*	22,750 (37.3)	134,802 (38.5)	<0.001 ^b^
*Females*	38,152 (62.7)	215,034 (61.5)	
Nursing triage code, n (% by column)			
*White*	8,869 (12.2)	56,656 (13.9)	<0.001 ^b^
*Green*	48,718 (70.0)	281,607 (67.7)	
*Yellow*	13,244 (18.2)	69,915 (16.5)	
*Red*	1,907 (2.6)	8,431 (1.9)	
Age group, n (% by column)			
*0 to 14 years*	15,166 (20.9)	77,062 (18.5)	<0.001 ^b^
*15 to 24 years*	5,628 (7.7)	35,124 (8.4)	
*25 to 64 years*	30,941 (42.5)	189,994 (45.6)	
*65 to 74 years*	7,041 (9.7)	40,488 (9.7)	
*75 or more years*	13,975 (19.2)	74,039 (17.8)	

^a^ Student t-test; ^b^ Chi-square test.

**Table 2.** Key performance indicators monitored during the study periods.

	December 2016 to January 2017	Other months of the windows period	*p*-value
Waiting times, median in minutes (IQR)			
*White*	52.0 (22.0–107.0)	45.0 (18.0–97.0)	<0.001 ^a^
*Green*	51.0 (19.0–116.0)	42.0 (16.0–96.0)	<0.001 ^a^
*Yellow*	21.0 (11.0–47.0)	18.0 (10.0–36.0)	<0.001 ^a^
*Red*	8.0 (4.0–15.0)	7.0 (4.0–13.0)	0.009 ^a^
Length of visit, median in minutes (IQR)			
*White*	10.0 (4.0–59.0)	12.0 (4.0–67.0)	0.0029 ^a^
*Green*	76.0 (12.0–152.0)	75.0 (13.0–148.0)	0.24 ^a^
*Yellow*	196.0 (108.5–380.0)	183.0 (102.0–351.0)	<0.001 ^a^
*Red*	212.0 (104.0–445.8)	184.0 (91.0–402.0)	<0.001 ^a^
Discharge from ED, n (% by row)			
*At home*	58,393 (80.3)	281,853 (81.9)	<0.001 ^b^
*Hospitalized*	9,658 (13.3)	42,155 (12.2)	<0.001 ^b^
*Abandonment*	3,247 (4.46)	14,273 (4.15)	0.0001 ^b^
*Dead*	200 (0.27)	578 (0.17)	<0.001 ^b^

^a^ Wilcoxon test; ^b^ Chi-square test.

**Table 3.** Multivariable logistic regression analysis on risk of death at discharge from ED.

	OR (adjusted by gender)	95% CI	*p*-value
Age in years (as continuous)	1.049	1.043–1.055	<0.001
Triage (“green” as reference)^§^			
*Yellow*	1.45	1.09–2.32	<0.001
*Red*	4.4	2.8–6.9	<0.001
December 2016 to January 2017 (other months as reference)	1.16	0.98–1.38	0.075

^§^ No death occurred among white code patients and thus they were excluded from the analyses.

## 4. Discussion

The present study was carried out in one of the most populous local health unit of Italy in order to investigate reasons contributing to ED service disruptions observed during 2016–2017 influenza season. The study, at our best knowledge, in our Country represents the first attempt to investigate performance of ED, especially during overcrowded phases. In Italy, January and December are usually critical periods for National Health Service since there is an overlapping of cold season, influenza epidemic and circulation of several respiratory pathogens. The important link between ED activities and the general population health is easily evincible by considering the epidemiological trend of hospitalization rate, influenza-like illness incidence and all-causes mortality in the general population [9]–[10]. In the Italian population, all these outcomes show peaks in the period during the which ED are overcrowded, highlighting the strict association between all these factors. In particular, different authors have observed that influenza can increase ED admissions, rising the risk of absenteeism among healthcare personnel and incrementing the burden of diseases in intensive care units as well as in other guards [11]–[13]. Muscatello et al estimated that influenza can be associated with approximately 1 in every 100 ED visits and more than 1 in 10 respiratory or infection visits, on average, concentrated across mid-winter to early spring [14]. Some authors have reported that influenza season can also have significant consequences in increasing the workload in the ED [15]. However there is the suspicion that influenza illness is not the only responsible for ED overcrowding and that other factors, as an aging population with a higher proportion of frail and chronic patients, could contribute in the next decades to increase the number of old patients seeking emergency care. In this sense, it seems that in Italy a primary role could currently played by a lack of access to primary care services and, overall, by an inadequate development of continuing care, especially for a group of patients described as “frail” [16].

Our results confirm the high health impact of the cold period (December and January) by determining an increase in ED admissions especially for older and critical/very critical patients (yellow and red triage codes). A not negligible consequence of these prolonged increments of activity is the lowering of ED services quality and patient safety. In this sense, it has been demonstrated that ED crowding can be associated with poorer quality of care and poorer health outcomes, along with extended waits for care [17]. Similar findings are consistent with those found in our study. During overcrowded periods we have observed an increase in waiting times, length of visits (for all triage codes but green code patients), percentage of hospitalization or abandonment and, odds of inpatient death.

International studies have shown that where a patient is waiting in the ED beyond the time the decision is made to admit, clinical outcomes are adversely affected [2]. In particular, long ED waiting and turnaround times have been shown to decrease both quality outcomes and patient satisfaction. Several studies have also demonstrated that reducing admit wait times can relieve ED congestion by decreasing ED length of stay, ambulance diversion hours, patients who abandon ED and costs [18]–[22]. In our area, waiting times increased especially for white and green code patients, who usually represent 80% of all ED admissions, accounting for a small but not negligible time increase (on average, about 7 and 9 minutes, respectively).

Overcrowding has had important consequences also on ED length of visits although this increase was particularly evident for yellow and red code patients. Probably, this last finding could be explained considering the higher number of complex patients admitted between December 2016 and January 2017. This hypothesis is also supported by the higher percentage of hospitalizations and older patients that have been admitted in ED during the overcrowded periods. In UK, in 2004 the proportion of patients presenting to ED who were 70 years or older was 198% higher than in 1990, and the proportion of patients 90 years or older was 671% higher [23]. The elderly were more often admitted to the hospital and once admitted had a greater length of stay compared to younger patients, an effect attributed to acute exacerbations of chronic diseases that require emergent care. Higher length of visits could be also explained as consequence of hospital bed shortage that is often associated with nursing staff availability, nursing ratios, ancillary service availability, local structure and likely many other factors.

Finally, several authors suggest that overcrowded months can increase inpatient mortality [24]. Similar findings were observed by other researchers suggesting a significant and variable association between measures of ED crowding and mortality (adjusted risk ratios ranging from 1.3 to more than 3) [24]–[26]. In our study we found an inpatient mortality risk increase of about 16%, although this result was marginally significant (*p* = 0.075) after adjustment for age, sex and admission triage codes. However, it should be noted that we identified overcrowdings using as proxy two months (December 2016 and January 2017) during the which patients and ED reported significant disruptions of the services. For this reason we cannot exclude that the presence of day misclassification (e.g. inclusion of not overcrowded days in December 2016 and January 2017) could have led to underestimation of association with reduction of precision and accuracy of estimates. This last represents a major limitation of our study. A second major limit was the lack of data for quantifying the possible association between outcomes and staff shortage, bed unavailability and/or delays in lab reports or delays in getting imaging results. Finally we have to consider that waiting times observed for very urgent patients (especially red code patients) could be recorded with some delay overestimating real waiting times. In conclusion our study highlights that ED overcrowding, especially during periods with increased morbidity as influenza season, can determine measurable worsening in ED services and patient outcomes as mortality, waiting times, lengths of stay, percentage of abandonment without being seen and, probably, costs.

Thus, address ED crowding has to be considered an important public health priority requiring policymakers involvement.
